# Insufficient Sampling to Identify Species Affected by Turbine Collisions

**DOI:** 10.1002/jwmg.852

**Published:** 2015-03-05

**Authors:** Julie A Beston, Jay E Diffendorfer, Scott Loss

**Affiliations:** U.S. Geological Survey, Geosciences and Environmental Change Science Center, Denver Federal Center MS 980Denver, CO, 80225, USA; Oklahoma State University, Natural Resource Ecology and Management005AC Ag Hall, Stillwater, OK, 74078, USA

**Keywords:** avian, fatality, monitoring, North America, turbine collisions, wind energy

## Abstract

We compared the number of avian species detected and the sampling effort during fatality monitoring at 50 North American wind facilities. Facilities with short intervals between sampling events and high effort detected more species, but many facilities appeared undersampled. Species accumulation curves for 2 wind facilities studied for more than 1 year had yet to reach an asymptote. The monitoring effort that is typically invested is likely inadequate to identify all of the species killed by wind turbines. This may understate impacts for rare species of conservation concern that collide infrequently with turbines but suffer disproportionate consequences from those fatalities. Published 2015. This article is a U.S. Government work and is in the public domain in the USA.

Wind power is rapidly expanding in the United States, with more electric generating capacity added than any other source in 2012 (Wiser and Bolinger 2013). Some stakeholders have raised concerns about fatalities caused when birds collide with wind turbines, and 1–2 years of post-construction monitoring are undertaken at many wind facilities to collect information on fatalities caused by turbine collisions. Over 220 species of birds have been collectively documented in fatality monitoring at wind facilities in North America (Loss et al. 2013). Most of these species fall under some level of protection from treaties and laws, such as the Convention for the Protection of Migratory Birds between the United States and Great Britain (acting on behalf of Canada), the United States Endangered Species Act, the Canadian Species at Risk Act, and the United States Bald and Golden Eagle Protection Act.

Ultimately, understanding the impacts of wildlife collisions with wind turbines requires knowledge of what species are killed by turbines, how many individuals are killed per year, and the consequences of those fatalities to the populations of species killed. Guidelines from the United States Fish and Wildlife Service (USFWS) state specifically that post-construction monitoring should “estimate the number and species composition of fatalities” (USFWS 2012:34). The majority of studies, development of statistical estimators, and reviews of wind–wildlife interactions have focused on the estimation of the number of fatalities. However, most studies of fatalities caused by wind turbines ultimately group data across species to estimate overall mortality rates by taxonomic groups (Johnson et al. 2002, Arnett et al. 2008, Loss et al. 2013). These studies may be useful in comparing relative fatality levels at different sites or across different causes of mortality (e.g., fossil fuel vs. wind energy generation; Government Accountability Office 2006, Sovacool 2009), but their utility is limited by the lack of species-specific information.

Several nonexclusive reasons may explain why species are not found or are found infrequently during fatality monitoring. Some species may effectively avoid turbines, and these species are unlikely to experience population-level effects of turbine fatalities. Other species may be infrequent in fatality monitoring because they are rare. Rarity is a problem for many species of conservation concern. Because they are rare, fatalities are infrequent, and therefore, difficult to detect, but those deaths may have disproportionately large impacts on populations. For example, the cerulean warbler (*Setophaga cerulean*) experienced severe declines during the 20th century and is considered vulnerable by the International Union for Conservation of Nature (Buehler et al. 2013). Although only 2 cerulean warbler fatalities were documented in publicly available monitoring data (Loss et al. 2013), those observations may represent a nontrivial stressor on populations of this struggling species. Finally, species may be undersampled.

We examined the relationship between sampling effort and the number of species observed dead at wind facilities in North America to assess the ability of fatality monitoring studies to accurately describe the community of avian species affected. Many approaches exist to estimate species richness from observation data (e.g., Gotelli and Colwell 2001, Dorazio et al. 2006), and many are applicable to fatality monitoring. Although these approaches can indicate how many species were likely missed, there is no way to determine which particular species were missed without further sampling. Hull et al. (2013) found the species accumulation curve for birds killed by collisions at 2 wind facilities in Tasmania began to asymptote after approximately 7 years of monitoring. Most studies in North America are of a shorter duration, suggesting these studies may not be capable of detecting the full range of species actually killed. Nevertheless, a wide body of literature on species accumulation curves and sampling effort suggests longer studies of a large number of turbines with short sampling intervals will detect more species than small, short studies with longer sampling intervals (Gotelli and Colwell 2001), and further analysis of how fatality studies accumulate species is warranted.

## Methods

We used data from 1995 to 2011 that were aggregated for a study of North American turbine fatalities (Loss et al. 2013) to assess the effect of effort on the number of species detected in wind turbine fatality monitoring. Our unit of interest was the wind facility, and we aggregated data from multiple studies at the same wind facility, including studies that sampled only 1 section or phase of the wind facility. For each facility, we calculated the total number of turbine-months of sampling effort, which was defined as the sum of the number of searched turbines multiplied by the study duration in months. We then modeled the total number of unique species documented at that wind facility across all studies and years as a logarithmic function of sampling effort. To correct for species richness, we repeated this using the percentage of species known to occur in or migrate through the state that were observed during fatality monitoring as the independent variable because site-specific estimates of richness were not feasible. We used state lists because they appeared to be the most inclusive community-level data available. For example, Breeding Bird Survey data poorly samples nocturnal species and arctic breeders. We ignored fatalities that could not be identified to species. Because turbine-months fail to incorporate differences in search intervals, we categorized each wind facility as having a short search interval (1–7 days) or a long search interval (>7 days) and compared species accumulation between these interval lengths. We selected the search intervals because surveys typically occurred either weekly or monthly for most facilities.

We also created species accumulation curves at 2 wind facilities to better characterize the patterns of accumulation at single wind facilities. Both Shiloh I Wind Power Project in Solano County, California (Kerlinger et al. 2007) and Wolfe Island Wind Plant in Frontenac Islands, Ontario (Stantec 2010, 2011*a*–*c*, 2012) had short sampling intervals (3–7 days), were monitored for at least 1 year, and reported the dates and species of avian fatality observations. This data structure allowed us to aggregate fatalities into weekly samples and produce species accumulation and sample-based rarefaction curves for each wind facility. We also used the Chao 1 method to calculate the expected number of species for each wind facility (Gotelli and Colwell 2011).

## Results

**Figure 1:**
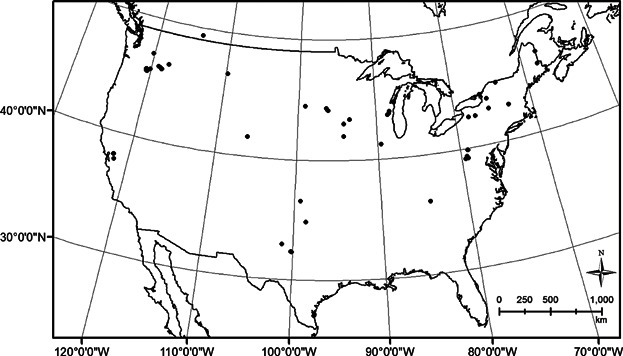
Locations of wind energy facilities used to estimate the relationship between mortality monitoring effort and the number of bird species observed.

**Figure 2:**
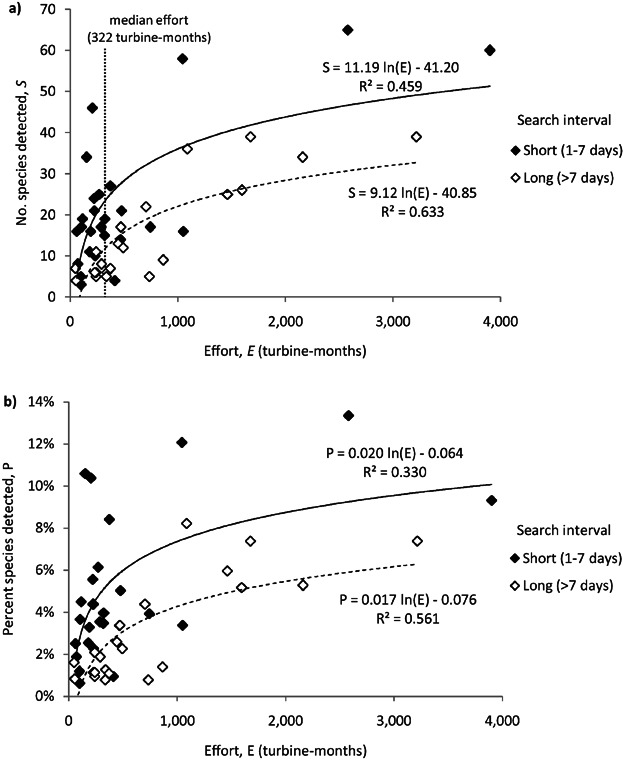
Number of species (a) and (b) percent of birds occurring in the state detected during fatality monitoring at 50 North American wind facilities sampled between 1995 and 2011 based on sampling interval and the sampling effort (number of turbines multiplied by the duration of study in months). The lines describe the relationships between species and effort for studies with short sampling intervals (solid) and long sampling intervals (dashed).

We calculated total turbine-months and number of species observed for 50 North American wind facilities (Fig. 1). Sampling effort explained 46 and 63% of the variation in the number of species and 33 and 56% of the variation in the percent of species detected at wind facilities studied with short and long sampling intervals, respectively (Fig. 2). Facilities that had greater effort or short sampling intervals detected more species than wind facilities with low effort or long sampling intervals (Fig. 2).

**Figure 3:**
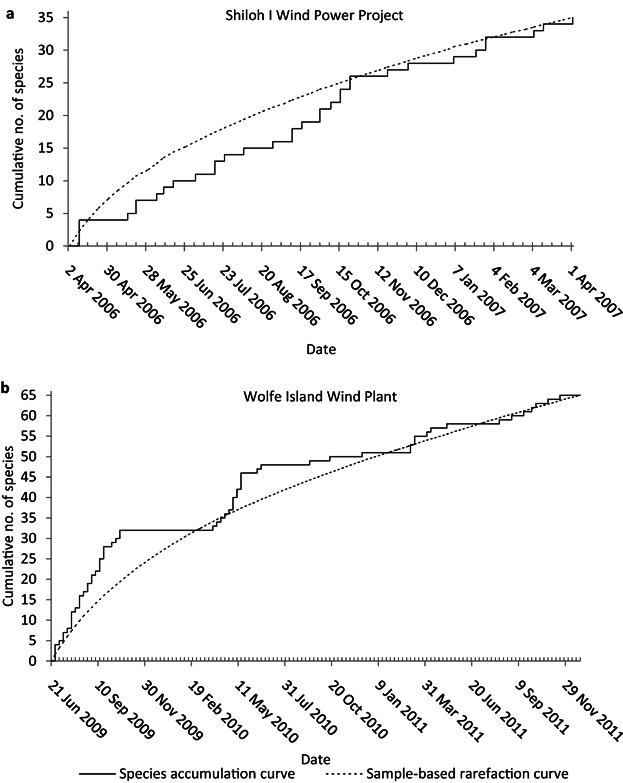
Species accumulation (solid) and sample-based rarefaction (dashed) curves for (a) a 52-week study at 50 turbines at Shiloh I Wind Power Project in Rio Vista, California, and (b) a 131-week study at 86 turbines at Wolfe Island Wind Plant in Frontenac Islands, Ontario.

Weekly surveys of 50 turbines at Shiloh I Wind Power Project accumulated species at a relatively constant rate through 52 weeks of monitoring (Fig. 3a). In contrast, weekly surveys at 86 turbines at Wolfe Island Wind Plant accumulated species quickly at the initiation of monitoring and in early spring each year (Fig. 3b). We estimated 66 (SD = 14) species were killed by Shiloh I turbines, with 35 observed, and 131 (14) species were killed by Wolfe Island turbines, with 65 observed. Furthermore, neither Shiloh I nor Wolfe Island appeared to have reached an asymptote after 1 and 2.5 years of study, respectively.

## Discussion

The wind facilities we included varied in species richness, abundance of species, number of turbines, proportion of turbines sampled, area, habitat types, sampling methodology, time of year studied, and other factors. Despite this latent variation in the data, there was still a clear relationship between effort and the number of species detected. This relationship remained when we attempted to correct for species richness using state and province species lists. Most of the wind facilities appeared to be undersampled with respect to species detection, with relatively low sampling effort and few species detected (Fig. 2). We note that the data we used came from publicly available studies and may not reflect the levels of sampling effort that exist in privately collected fatality studies. The patterns of accumulation at Shiloh I Wind Power Project and Wolfe Island Wind Plant suggest that sampling >2 years may be warranted to detect all affected species. Moreover, the faster rates of species accumulation during early spring at Wolfe Island highlight the importance of covering migratory periods during fatality studies.

Generally, studies with weekly (or more frequent) sampling detected more species than studies with longer sampling intervals. Long periods between sampling make it more likely that any new species that is killed will be removed by scavengers or otherwise degrade to anonymity before it can be observed. Although applying a correction factor for scavenger removal can reduce the resulting bias in estimates of fatalities across taxonomic groups (Smallwood 2013), it cannot indicate the identities of those individuals that went undetected. Thus, a study that is sufficient to estimate the number of fatalities caused by turbines could still be insufficient to determine which species are affected. The proportions of each species in fatality data multiplied by the total fatalities were used to calculate species-specific fatality levels for birds colliding with communication towers (Longcore et al. 2013) and wind turbines (Zimmerling et al. 2013), but this approach is likely to produce inaccurate estimates for wind fatality data where the species composition of fatalities is not fully described.

## Management Implications

Though fatality estimates provide useful information about wildlife impacts of wind energy production, managers cannot assess risks to populations or species without characterization of the identities of the animals that are killed. Available fatality data were insufficient to conclude that species not observed were not in fact affected by turbine fatality. Even the studies with short sampling intervals and long study duration at Shiloh and Wolfe Island were unable to determine with confidence that all affected species of conservation concern were identified. In light of these limitations, managers may need to seek alternate methods or additional data when addressing rare species of conservation concern at wind facilities. For example, it may be possible to estimate the pool of species at risk, or rare species, that will likely occur around a facility (and thus may be killed but rarely found) using state lists, or combinations of data from monitoring programs.
